# Association between eating behaviors and premenstrual syndrome severity among Japanese female university students: a cross-sectional study

**DOI:** 10.7717/peerj.21141

**Published:** 2026-04-23

**Authors:** Yuna Akase, Yuka Yoshinari, Miko Fujimori, Otoha Yoshioka, Momoko Nagai-Tanima, Tomoki Aoyama

**Affiliations:** Department of Physical Therapy, Human Health Sciences, Graduate School of Medicine, Kyoto University, Kyoto, Japan

**Keywords:** Premenstrual syndrome, Eating behavior, Eating attitude, University students

## Abstract

**Background:**

Premenstrual syndrome (PMS) affects the quality of life (QOL) and daily functioning of young women. Dietary and nutritional intake are a suggested approach to alleviate PMS symptoms However, lifestyle factors of female students, such as living alone or dietary restrictions, may exacerbate PMS symptoms. This study investigated the relationship between PMS symptoms and eating behaviors among female university and graduate students, identifying dietary behavior factors that may exacerbate or alleviate these symptoms.

**Methods:**

An online survey was targeted female university and graduate students aged 18 to under 25. The questionnaire included basic information, PMS symptoms, and eating behaviors. PMS symptoms were assessed using the Premenstrual Syndrome (PMS/PMDD) Questionnaire. Eating behaviors were evaluated using the Eating Behavior Questionnaire. Eating behavior scores across PMS severity groups were compared using one-way Analysis of Variance (ANOVA) followed by Tukey–Kramer tests.

**Results:**

Among the 164 participants, the “Severe PMS group” had a significantly higher “Total” score (*p* = 0.04) than the “Moderate PMS” (Cohen’s d = 0.907) and the “No/Mild PMS” (Cohen’s d = 0.886) groups. And the “Severe PMS group” had a significantly higher sub-score “Hunger and satiety” on the Eating Behavior Questionnaire than the “Moderate PMS” (Cohen’s d = 1.039) and the “No/Mild PMS” (Cohen’s d = 0.915) groups.

**Conclusion:**

PMS severity was associated with eating behaviors, particularly hunger and satiety, suggesting that foods that promote fullness or stabilize blood sugar levels may help alleviate PMS symptoms. Addressing eating behaviors, alongside nutritional intake, may be an effective approach to managing PMS symptoms. However, establishing a causal relationship between these factors requires longitudinal research.

## Background

Premenstrual syndrome (PMS) consists of distressing symptoms during the luteal phase of the menstrual cycle, impairing the patient’s normal daily functioning and resolving immediately after menstruation begins. The luteal phase starts after ovulation and ends with the onset of menstruation (Hofmeister & Bodden, 2016). PMS includes psychological (depression and mood instability) and physical symptoms (abdominal pain, breast tenderness, headache, and fatigue (Dickerson, Mazyck & Hunter, 2003). When sever psychological symptoms dominate, PMS is classified as premenstrual dysphoric disorder (PMDD), a more serious condition characterized by impairments in daily functioning and relationship breakdown (Braverman, 2007).

PMS affects 80.5% of university students experiences (Akın & Erbil, 2024), with high incidence in late teens to early twenties (Hashim et al., 2019), emphasizing the need for appropriate support and information dissemination. Among female college students aware of PMS symptoms, 60.4% reported interference with their normal lives, and 35.4% missed school or work (Mohib et al., 2018). Moreover, college students with mild PMS had lower quality of life (QOL) scores than non-PMS students (Victor et al., 2019). PMS symptoms impact the lives and QOL of young women, making it a critical issue.

Various studies associate PMS prevalence with lifestyle habits such as lack of regular exercise (Bhuvaneswari, Rabindran & Bharadwaj, 2019) and decreased sleep quality (Cheng et al., 2013). Nutrient intake adjustments are recommended for PMS symptom management, with a balanced diet as the first step in PMS treatment (Jefferson, 2003). Higher vitamin D (Bertone-Johnson et al., 2005) and zinc (Jafari, Amani & Tarrahi, 2020) intake are associated with decreased PMS risk. High fat (Hashim et al., 2019) and fast food (Thakur et al., 2022) intake are associated with an increased PMS risk, suggesting that diet and PMS symptoms are closely related. Unhealthy eating behaviors, such as emotional eating, restrictive dieting, and meal skipping, may alter nutrient intake patterns and contribute to PMS symptoms, particularly as these behaviors fluctuate across the menstrual cycle (Klump et al., 2013).

Female students often equate body image perceptions of thinness with beauty, leading to excessive dieting and disordered eating behaviors (Aparicio-Martinez et al., 2019). Furthermore, living alone during higher education often leads to eating behavior issues, including nutrient imbalances, irregular mealtimes, and increased reliance on eating out, contributing to weight issues (Kibayashi & Nakade, 2024). This lifestyle transition significantly influences students’ dietary choices and eating patterns, often leading to intake of less healthy foods and meal skipping (Papadaki et al., 2007). Furthermore, thinness and obesity are associated with PMS symptom severity (Park et al., 2022). We have also identified a relationship between eating disturbance and PMS in previous studies (Yoshinari et al., 2024). Despite this research on nutrition and eating disturbances, the link between PMS and a wide range of eating behaviors remains unclear.

Herein, we hypothesized that eating behaviors, in addition to nutrient intake, are associated with PMS symptoms. This study aimed to investigate the association between PMS symptoms and eating behaviors among female university and graduate students using the primary assessment and subscales of the Eating Behavior Questionnaire in a cross-sectional manner. The goal is to identify eating behavior factors that may exacerbate or alleviate these symptoms. Accordingly, the present study was conducted to test this hypothesis and provide insights into how eating behavior patterns may influence PMS symptom severity in this population.

## Methods

### Participants and procedure

This was a cross-sectional observational study conducted in Japan. Inclusion criteria were female undergraduate and graduate students aged 18 to under 25. The exclusion criterion was individuals with psychiatric disorders unrelated to PMS. An online questionnaire was conducted, with informed consent. After providing sufficient explanation, the patient pressed the acceptance button, thereby granting informed consent.

The questionnaire was created using a web-based survey service, Survey Monkey (San Mateo, CA, USA), and cooperation requested *via* social networking services. The questionnaire included basic information (age, height, and weight), PMS symptoms, and eating behaviors.

Participants classified as having PMDD based on their responses were not provided with referrals or support. However, individual feedback on questionnaire results was offered upon participant request.

### Ethics declarations

#### Ethics approval and consent to participate

This study was approved by the Ethics Committee of the Graduate School of Medicine, Faculty of Medicine, Kyoto University, and its affiliated hospitals (approval number: R3665-2). The study was fully explained online, and informed consent was obtained upon questionnaire submission. This study was conducted in accordance with the Declaration of Helsinki and Japanese ethical guidelines for medical and biological research involving human participants.

### Questionnaire

PMS symptoms severity was evaluated using “The Premenstrual Symptoms Screening Tool (PSST)” (Kamada, 2012), a partially modified and Japanese-translated version of the questionnaire developed by Steiner, Macdougall & Brown (2003) (Table S1). Although the Daily Record of Severity of Problems (DRSP) remains the gold standard for PMS diagnosis, this method requires participants to take detailed daily records over two menstrual cycles, meaning it can be burdensome in clinical practice (Kamada, 2012). We instead selected the PSST for its simplicity and practicality as a retrospective screening tool. The “Japanese version of the PSST” consists of 18 items on symptoms experienced from 1 week before to during menstruation, and five items related to daily life impact. The self-reported symptoms and daily life impact were assessed on a four-point scale: “Not at all”, “Mild”, “Moderate”, and “Severe”. Participants were classified into four groups based on PMS severity: “PMDD group”, “Severe PMS group”, “Moderate PMS group”, “No/Mild PMS group”, according to diagnostic criteria. This classification approach is consistent with previous studies that have similarly used similar four-group divisions based on PMS severity. Separating PMDD for Severe PMS allows for a clearer distinction in symptom severity and associated outcomes.

Eating behavior was evaluated using the Eating Behavior Questionnaire (The Japan Society for the Study of Obesity Committee for the Guidelines for the Management of Obesity Disease, 2022; Tsukamoto-Kawashima et al., 2024), which was used to assess eating behaviors in patients with obesity. The questionnaire, consisting of 55 items, quantifies deviations and tendencies in eating behaviors, with higher scores indicating greater problematic eating behaviors (Table S2). Each question is answered on a four-point scale: “1 point: Not at all”, “2 points: Occasionally true”, “3 points: Often true”, and “4 points: Exactly true”. The total score and scores for seven subcategories—“Knowledge of body constitution and weight”, “Motive for eating”, “Feeding by proxy”, “Hunger and satiety”, “Eating patterns”, “Meal plan”, and “Eating habits”—were calculated.

The authors have permission to use this instrument for the copyright holders. The Japanese version of the EBQ has also been officially adopted in Guidelines for the Management of Obesity Disease 2022, and has since been applied in both clinical and epidemiological studies in Japan (*e.g.*, Tsukamoto-Kawashima et al., 2024; Fujii et al., 2025). Although it has not yet been formally validated, it is recognized as a standard tool for assessing eating behaviors in the Japanese context. Internal consistency of the Eating Behavior Questionnaire was assessed using Cronbach’s alpha. The total scale showed excellent internal consistency (Cronbach’s α = 0.91), and the seven subscales also demonstrated good reliability (Cronbach’s α = 0.81).

### Statistical analyses

Participants were divided into four groups based on PMS severity: “PMDD group”, “Severe PMS group”, “Moderate PMS group”, and “Mild/No PMS group”. The mean total scores of the Eating Behavior Questionnaire were calculated for each group. Subsequently, the Shapiro–Wilk test was performed, followed by one-way analysis of variance. Multiple comparison tests (Tukey–Kramer’s HSD test) were conducted for items with significant differences.

Statistical analyses were performed using JMP Pro 16 (SAS Institute Inc., Cary, NC, USA), with the significance level set at less than 5%

## Results

The survey received responses from 236 participants. Among these, responses that were incomplete or outside the scope of the study were excluded. Consequently, 164 valid responses were included in the analysis (valid response rate: 69.5%).

Among the 164 participants, 12 (7.3%) were classified in the “PMDD group”, 10 (6.1%) in the “Severe PMS group”, 53 (32.3%) in the “Moderate PMS group”, and 89 (54.3%) in the “No/Mild PMS group”. No significant differences in baseline characteristics were observed among the four groups (Table 1).

**Table 1:** Comparison of basic characteristics across PMS severity groups.

n = 164	**PMDD** 7.3% (*n* = 12)	**Severe PMS** 6.1% (*n* = 10)	**Moderate PMS** 32.3% (*n* = 53)	**No/Mild PMS** 54.3% (*n* = 89)	*P* value
Age (year)	21.3 ± 1.8	20.3 ± 1.5	21.0 ± 1.7	20.8 ± 1.6	0.50
Height (cm)	160.4 ± 5.5	160.5 ± 3.8	158.6 ± 5.6	159.1 ± 4.9	0.58
Weight (kg)	53.2 ± 7.3	52.8 ± 8.5	49.8 ± 5.0	51.0 ± 6.1	0.22
BMI (kg/m^2^)	20.6 ± 2.2	20.5 ± 3.3	19.8 ± 1.6	20.1 ± 2.1	0.49

**Notes.**

PMS premenstrual syndrome PMDD premenstrual dysphoric disorder BMI body mass index

Mean ± standard deviation.

**Figure 1:**
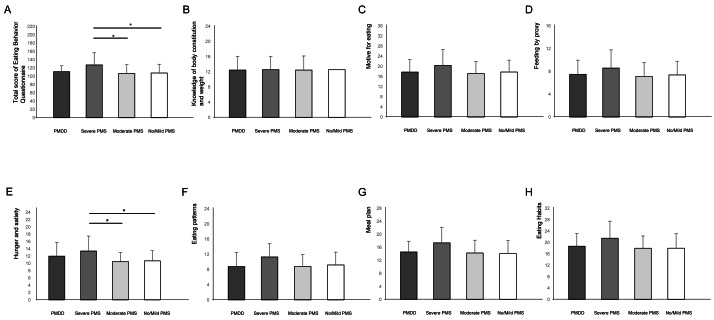
Comparison of Eating Behavior Questionnaire across PMS severity groups. Total score and subscores on the eating behavior questionnaire across PMS severity groupswas compared across PMS severity groups. (A) Total Score and subscores on the Eating Behavior Questionnaire. (B) Knowledge of body constitution and weight. (C) Motive for eating. (D) Feeding by proxy. (E) Hunger and Satiety. (F) Eating patterns.

The Eating Behavior Questionnaire total scores were compared across the four PMS severity groups (Fig. 1A). Overall, the average total score on the Eating Behavior Questionnaire (Table 2) was significantly higher in the Severe PMS group (127.4 ± 29.3) than in the Moderate PMS (106.9 ± 21. 3) (*p* = 0.03, Cohen’s *d* = 0.91, 95% CLs [0.21–1.60]) and the No/Mild PMS (108.0 ± 21.0) (*p* = 0.04, Cohen’s *d* = 0.89, 95% CLs [0.22–1.55]) groups.

**Table 2:** Comparison of total scores on the eating behavior questionnaire across the PMS severity groups.

	**PMDD** (*n* = 12)	**Severe PMS** (*n* = 10)	**Moderate PMS** (*n* = 53)	**No/Mild PMS** (*n* = 89)	*P* value
Mean ± SD	111.1 ± 14.6	127.4 ± 29.3^†^^‡^	106.9 ± 21.3^†^	108.0 ± 21.0^‡^	0.03^*^
95% CLs	98.95–123.22	114.11–140.69	101.17–112.72	103.56–112.47	
Median (25%–75% quartiles)	110.0 (107.0–113.25)	118.0 (109.0–142.5)	104.0 (95.0–120.0)	107.0 (92.0–120.0)	

**Notes.**

PMS premenstrual syndrome PMDD premenstrual dysphoric disorder SD standard deviation CLs Confidence Levels

Multiple comparison tests (Tukey–Kramer’s HSD test) were conducted for items with significant differences.

† Severe PMS *vs* Moderate PMS (*p* = 0.03).

‡ Severe PMS *vs* No/Mild PMS (*p* = 0.04).

* 1 < 0.05.

The Eating Behavior Questionnaire “Subcategories” scores were compared across the four PMS severity groups (Figs. 1B–1H, Table 3).

**Table 3:** Comparison of “subcategories” scores on the eating behavior questionnaire across the PMS severity groups.

		**PMDD** (*n* = 12)	**Severe** **PMS** (*n* = 10)	**Moderate PMS** (*n* = 53)	**No/Mild** **PMS** (*n* = 89)	*P*value
Knowledge of body constitution and weight	Mean ± SD	12.5 ± 3.6	12.6 ± 3.5	12.5 ± 3.7	12.6 ± 3.5	0.56
	95% CLs	10.2–14.8	11.4–17.0	11.4–13.5	11.8–13.3	
	Median (25%–75% quartiles)	12.0 (9.75–14.5)	14.0 (11.5–16.0)	12.0 (10.0–15.0)	12.0 (10.0–15.0)	
Motive for eating	Mean ± SD	17.8 ± 4.9	20.4 ± 6.3	17.2 ± 4.8	17.8 ± 4.7	0.29
	95% CLs	14.7–21.0	15.9–24.9	15.8–18.5	16.8–18.8	
	Median (25%–75% quartiles)	17.5 (14.5–20.25)	19.5 (16.0–23.75)	16.0 (13.0–20.0)	17.0 (15.0–21.0)	
Feeding by proxy	Mean ± SD	7.5 ± 2.5	8.6 ± 3.2	7.1 ± 2.5	7.4 ± 2.4	0.38
	95% CLs	5.9–9.1	6.3–10.9	6.4–7.8	6.9–7.9	
	Median (25%–75% quartiles)	7.5 (5.75–9.25)	8.0 (6.25–10.5)	7.0 (5.0–9.0)	7.0 (5.0–9.0)	
Hunger and satiety	Mean ± SD	12.0 ± 3.8	13.4 ± 4.1^†^^‡^	10.5 ± 2.5^†^	10.7 ± 2.8^‡^	0.02^*^
	95% CLs	9.6–14.4	10.4–16.4	9.9–11.2	10.1–11.3	
	Median (25%–75% quartiles)	12.0 (8.75–14.25)	13.0 (10.25–166.5)	10.0 (9.0–12.0)	10.0 (9.0–12.0)	
Eating patterns	Mean ± SD	8.8 ± 3.7	11.3 ± 3.5	8.8 ± 3.2	9.2 ± 3.4	0.20
	95% CLs	6.5–11.1	8.8–13.8	8.0–9.7	8.5–9.9	
	Median (25%–75% quartiles)	8.5 (5.75–11.25)	11.0 (8.5–12.0)	8.0 (6.0–11.0)	9.0 (6.0–12.0)	
Meal plan	Mean ± SD	14.6 ± 3.3	17.4 ± 4.8	14.3 ± 3.9	14.1 ± 4.0	0.11
	95% CLs	12.5–16.7	14.0–20.8	13.3–15.4	13.3–15.0	
	Median (25%–75% quartiles)	13.5 (12.0–17.25)	18.0 (13.25–20.5)	14.0 (11.0–17.0)	14.0 (11.0–17.0)	
Eating habits	Mean ± SD	18.7 ± 4.5	21.5 ± 6.0	18.0 ± 4.3	18.0 ± 5.1	0.19
	95% CLs	15.8–21.5	17.2–25.8	16.8–19.2	16.9–19.1	
	Median (25%–75% quartiles)	18.0 (16.25–21.5)	23.0 (18.0–26.5)	18.0 (15.0–21.0)	17.0 (14.0–22.0)	

**Notes.**

PMS premenstrual syndrome PMDD premenstrual dysphoric disorder SD standard deviation CLs Confidence Levels

Multiple comparison tests (Tukey–Kramer’s HSD test) were conducted for items with significant differences.

* *p*  <  0.05.

† Severe PMS *vs*. Moderate PMS (*p* = 0.02).

‡ Severe PMS *vs*. No/Mild PMS (*p* = 0.03).

Overall, the average “Hunger and satiety” score on the Eating Behavior Questionnaire (Table 3) was significantly higher in the Severe PMS group (13.4 ± 4.1) (*p* = 0.02, Cohen’s *d* = 1.04, 95% CLs [0.34–1.74]) than in the Moderate PMS (10.5 ± 2.5) and No/Mild PMS (10.7 ± 2.8) (*p* = 0.04, Cohen’s = 0.92, 95%CLs [0.25–1.58]) groups. Scores in other subcategories also showed a tendency to be higher in the Severe PMS group than in the Moderate or No/Mild PMS groups, though these differences did not reach statistical significance (Fig. 1, Table 3).

## Discussion

This study aimed to identify dietary behavior factors that may exacerbate or alleviate PMS symptoms in female university and graduate students. The “Severe PMS group” had a significantly higher “Total” score on the Eating Behavior Questionnaire than the “Moderate PMS” and the “No/Mild PMS” groups. The “Severe PMS group” had a significantly higher “Hunger and satiety” score on the Eating Behavior Questionnaire than the other groups.

The “Severe PMS group” had a significantly higher average “Total” score on the Eating Behavior Questionnaire was in the compared to the “Moderate PMS” and the “No/Mild PMS” groups. These results suggested that the greater the severity of PMS, the more pronounced the eating behavior problems. Although PMS has been associated with nutrient and diet intake, studies indicate that eating behaviors, rather than the amount of food intake, are related to PMS (Isgin-Atici et al., 2018). Students with irregular breakfast habits, more than two cups of coffee per day, frequent alcohol or fast-food consumption tend to have higher PMS scores (Isik et al., 2016). Additionally, the quality of university life is significantly reduced as PMS severity increases. These findings suggest that PMS symptoms in university students are influenced by multiple dietary behavioral factors, such as irregular eating patterns (*e.g.*, skipping breakfast), excessive consumption of certain foods or stimulants, and stress-related disruptions in eating behavior. University students’ busy academic schedules and flexible lifestyles may amplify these risk factors, exacerbating PMS symptoms. Thus, there is a close relationship between PMS symptoms and eating behaviors. The risk of eating disorders increases with the severity of PMS symptoms (Çoban et al., 2021) and women experiencing severe PMS symptoms tend to have higher calorie intake (Giannini et al., 1985). However, several studies have found no significant association between dietary factors and PMS. For example, Houghton et al. (2018) and Farasati et al. (2015) both reported no clear link between PMS and intake of nutrients such as fiber, carbohydrates, protein, dairy products, caffeine, and vegetables. These discrepancies may stem from differences in study designs, dietary assessment methods, and participant characteristics. Despite these inconsistencies, the present study adds to the growing body of evidence suggesting a link between eating behaviors and PMS. Overall, these results suggest that abnormalities in eating behaviors are more likely to occur as PMS severity increases. Therefore, reviewing nutrient intake and overall eating behaviors may contribute to alleviating PMS symptoms.The “Severe PMS group” had a significantly higher average “Hunger and satiety” score on the Eating Behavior Questionnaire than the “Moderate PMS” and “No/Mild PMS” groups. These results suggest that the greater the severity of PMS, the more pronounced the eating behavior problems related to hunger and satiety. As physiological factors, hormones and neurotransmitters fluctuate throughout the menstrual cycle, with serotonin levels reaching their lowest before menstruation (Taylor et al., 1984). Patients with PMS experience an even greater serotonin decline (Ashby Jr et al., 1988), affecting appetite suppression, eating regulation, and satiety (Blundell, 1992), leading to overeating and cravings for carbohydrates (Fernstrom & Wurtman, 1971). This serotonergic dysregulation can impair appetite control and satiety, consequently leading to increased sensitivity to food cues and carbohydrate cravings in the premenstrual phase (Papadaki et al., 2007). In addition, hormones involved in appetite regulation, including leptin and ghrelin, may contribute to the observed alterations in hunger and satiety among individuals with severe PMS (Scheid & De Souza, 2010). Although these hormones were not directly measured in the present study, prior research has indicated that leptin and ghrelin levels fluctuate throughout the menstrual cycle and under conditions of energy deficiency, potentially exacerbating dysregulated eating behaviors. Additionally, during the luteal phase of the menstrual cycle, blood sugar levels fluctuate significantly (Pulido & Salazar, 1999), which is considered a factor contributing to an increased sense of hunger. In addition to these physiological factors, psychological factors are influence hunger and satiety in eating behaviors. Emotional eating is defined as “a tendency to overeat in response to negative emotions such as anxiety or irritation” (Van Strien et al., 2007). These behaviors occur independently of hunger signals (Adam & Epel, 2007) and may contribute to disruptions in eating behaviors. Furthermore, emotional eating behaviors tend to increase during the mid-luteal phase of the menstrual cycle, when PMS symptoms are most pronounced (Klump et al., 2013), and the severity of PMS may further intensify this tendency. Fluctuations in serotonin and blood sugar levels throughout the menstrual cycle likely influence hunger and satiety. Psychological factors such as mental stress and emotional instability also contribute to variations in eating behaviors. Eating behaviors related to hunger and satiety are closely tied to these fluctuations, suggesting a strong interaction between PMS symptoms and eating patterns. Therefore, it is proposed that consuming foods that help maintain satiety and stabilize blood sugar levels may effectively alleviate PMS symptoms. These results are consistent with the findings of a study conducted among nursing students in Turkey, which showed that emotional eating was significantly associated with PMDD symptoms, and that emotional and uncontrolled eating behaviors became more prominent as the severity of premenstrual symptoms increased (Dye & Blundell, 1997).

This study has several limitations. First, the cross-sectional design prevented establishing a causal relationship between PMS symptoms and eating behaviors. Second, PMS symptoms were evaluated using the PSST, a relatively simple screening tool using retrospective assessment, without any analysis of objective physiological data, such as hormone or blood glucose levels; this limits the assessment accuracy. Further, the DRSP is the gold standard for PMS diagnosis, rather than the PSST, and the Japanese version of the PSST has not yet been formally validated, as further studies are needed. Similarly, eating behaviors were assessed using the EBQ, a self-reported questionnaire officially adopted by the Japanese obesity guidelines; however, its formal validation in this population remains limited. Further, actual behaviors were not directly tracked, potentially reducing statistical power. In this survey, cases where not all questions on the questionnaire were answered were excluded as data missing. Mechanisms such as limiting the number of questions or establishing mandatory items are necessary to prevent data missing. Third, recruitment was conducted *via* social media, which may have introduced selection bias. Further, duplicate responses could not be fully eliminated due to platform limitations. Methods such as requiring respondent authentication or providing an email address can prevent duplicate responses, but these were not implemented in this survey. Fourth, physical activity, a known confounder of PMS, was not evaluated, possibly influencing the results. Finally, the generalizability of the findings may be limited, as all the participants were Japanese female undergraduate and graduate students. Overall, cultural, dietary, and lifestyle differences should be considered when applying these results to other populations. Future research should employ a prospective cohort study involving a larger sample of female undergraduate and postgraduate students, utilizing daily DRSP surveys and standardized dietary behavior assessments. At that time, sleep, stress, and living environment should also be assessed as parameters, and multivariate analysis should be conducted to clarify the contribution of key dietary behaviors more clearly. This approach could clarify causal relationships between eating behavior factors and PMS prevention or improvement. Furthermore, disseminating accurate knowledge about PMS symptoms and eating behaviors could promote prevention and awareness activities, potentially contributing to an improvement in women’s QOL.

## Conclusion

This study investigated the relationship between PMS symptoms and eating behaviors among female undergraduate and graduate students. Severe PMS symptoms were strongly associated with problematic eating behaviors, particularly those related to hunger and satiety subscale. The findings suggest that addressing these specific eating behaviors, particularly the regulation of hunger and satiety, may help alleviate PMS symptoms. Future studies need to clarify causal links with longitudinal designs and biological data. Clinically, supporting appetite-focused healthy eating may help to manage PMS in young women. Overall, these findings reveal a novel link between PMS severity and specific eating behaviors, guiding future research and care.

## Supplemental Information

10.7717/peerj.21141/supp-1 Supplemental Information 1 STROBE checklist

10.7717/peerj.21141/supp-2 Supplemental Information 2 Raw data

10.7717/peerj.21141/supp-3 Supplemental Information 3 Eating Behavior Questionnaire

## Additional Information and Declarations

### Data Availability

The following information was supplied regarding data availability: The raw data is available in the Supplemental Files.
