# Supplementary material for: Association between eating behaviors and premenstrual syndrome severity among Japanese female university students: a cross-sectional study
Source: PeerJ. 2026 Apr 23;14:e21141. doi: 10.7717/peerj.21141 (PMC13110648; doi:10.7717/peerj.21141)
Supplement: Supplemental Information 3 [file peerj-14-21141-s003.docx]

**Supplementary Table1** Eating Behavior Questionnaire

| Subcategories | items |
| --- | --- |
| **Knowledge of body constitution and weight** | Do you think you gain weight because you like sweets?  Do you think you gain weight because you lie down immediately after eating?  Do you eat well even when you have a cold?  Do you really think you gain weight even by drinking water?  Are you convinced you gain weight because of lack of exercise?  Do you feel that you are more prone to weight gain than others? |
| **Motive for eating** | Do you eat more than you need just to have no leftovers?  If your favorite food is offered, do you eat it even immediately after a meal?  Do you feel unsatisfied with dinner when there are only a few dishes?  Do you tend to eat when you see others eating?  Do you tend to order more food than needed when you eat out or have food delivered?  Do you feel unsatisfied unless you buy more groceries than needed?  Are you not satisfied unless you cook more food than needed?  Do you indulge in unplanned buying of food that looks delicious in a supermarket, etc.?  Do you often have meals just for social reasons? |
| **Feeding by proxy** | Do you feel restless when there is not much food in the refrigerator?  Do you tend to eat when you feel irritated or anxious?  Do you keep foods readily available?  Do you eat something or other when you have nothing else to do? |
| **Hunger and satiety** | Do you feel irritated when hungry?  Do you feel satiated only when your stomach is full?  Do you have regrets after eating too much?  Are you unable to sleep when you are hungry?  Do you really not have the feeling of hunger or fullness?  Are you often not hungry even just before a meal? |
| **Eating patterns** | Do you eat fast?  Are you often told that you eat too much?  Do you not chew your food well?  Do you cram food into your mouth when you eat?  During a meal do you stuff food items into your mouth one after another? |
| **Meal plan** | Do you often use convenience stores?  Do you like noodles?  Do you often eat out or have food delivered?  Do you often go to fast food joints and have burgers and the like?  Do you often eat sweet buns?  Do you like fatty foods?  Do you eat meat most of the time? |
| **Eating habits** | Do you have supper late in the evening?  Are you an evening person?  Do you tend to gain weight during consecutive holidays, the Bon (Japanese Buddhist holidays in August) and New Year holidays?  Do you snack often?  Are your meal times irregular?  Is supper the largest and most sumptuous of your meals?  Do you have supper late in the evening?  Do you skip breakfast? |

※7 sub-items plus 10 additional items, totaling 55 items.
